# Engineering Components of the *Lactobacillus* S-Layer for Biotherapeutic Applications

**DOI:** 10.3389/fmicb.2018.02264

**Published:** 2018-10-02

**Authors:** Courtney Klotz, Rodolphe Barrangou

**Affiliations:** ^1^Genomic Sciences Graduate Program, North Carolina State University, Raleigh, NC, United States; ^2^Department of Food, Bioprocessing and Nutrition Sciences, North Carolina State University, Raleigh, NC, United States

**Keywords:** *Lactobacillus*, probiotic, S-layer, biotherapeutic, mucosal vaccine, CRISPR

## Abstract

Lactic acid bacteria (LAB) are frequently harnessed for the delivery of biomolecules to mucosal tissues. Several species of *Lactobacillus* are commonly employed for this task, of which a subset are known to possess surface-layers (S-layers). S-layers are two-dimensional crystalline arrays of repeating proteinaceous subunits that form the outermost coating of many prokaryotic cell envelopes. Their periodicity and abundance have made them a target for numerous biotechnological applications. In the following review, we examine the multi-faceted S-layer protein (Slp), and its use in both heterologous protein expression systems and mucosal vaccine delivery frameworks, through its diverse genetic components: the strong native promoter, capable of synthesizing as many as 500 Slp subunits per second; the signal peptide that stimulates robust secretion of recombinant proteins; and the structural domains, which can be harnessed for both cell surface display of foreign peptides or adhesion enhancement of a host bacterium. Although numerous studies have established vaccine platforms based on one or more components of the *Lactobacillus* S-layer, this area of research still remains largely in its infancy, thus this review is meant to not only highlight past works, but also advocate for the future usage of Slps in biotherapeutic research.

## Introduction

Lactic acid bacteria (LAB) are Gram-positive, anaerobic or microaerophilic, non-sporulating microorganisms that inhabit diverse environments including milk and plant surfaces, as well as the mouth, gastrointestinal tract, and vaginal tract of humans and animals (Liu et al., 2014). Traditionally, they have been employed for food and dairy fermentations, but have more recently garnered attention for their health-promoting properties with many species used widely as probiotics (Klein et al., 1998; Klaenhammer et al., 2005). Recombinant LABs are frequently harnessed for mucosal delivery of biomolecules such as therapeutic proteins or vaccine antigens (Berlec et al., 2012). In comparison to traditional intravenous or intramuscular vaccine administration, the mucosal route enables immunizations to be performed orally, reducing potential side effects while increasing specificity for chronic illnesses and infections associated with mucosal tissues (Bermudez-Humaran et al., 2011; Wyszynska et al., 2015). Furthermore, many LABs are bile and acid tolerant, act as natural adjuvants, and interact with cells of the immune system (Wells and Mercenier, 2008), making them ideal candidates for antigen carriage.

The LABs most frequently chosen for vaccine delivery are *Lactococcus lactis* and select species of the *Lactobacillus* genus (Wells and Mercenier, 2008; Bermudez-Humaran et al., 2011, 2013; Wyszynska et al., 2015). However, unlike *L. lactis*, several species *Lactobacillus* have been shown to possess surface-layers (S-layers) (Hynonen and Palva, 2013). S-layers have been detected on both Gram-positive and Gram-negative bacteria and are nearly ubiquitous in archaea (Fagan and Fairweather, 2014). They are defined as two-dimensional crystalline arrays composed of repeating proteinaceous subunits that constitute the outermost layer of a cell envelope (Fagan and Fairweather, 2014). The S-layer proteins (Slps) attach to the underlying peptidoglycan via electrostatic interactions and possess inherent, entropy-driven affinities to self-assemble with each other (Hynonen and Palva, 2013). Thus far, S-layers have been characterized for their role in maintaining cell shape; acting as molecular sieves; serving as binding sites for large molecules, ions, or phages; and mediating surface adhesion (Sleytr et al., 2014). Additionally, Slps are some of the most abundant proteins synthesized by the cell, making them metabolically expensive but also underscoring their importance to the organism (Sara and Sleytr, 2000; Fagan and Fairweather, 2014). Their high expression, periodicity, and self-assembling properties have made them a target for numerous applications in biotechnology and nanotechnology (Avall-Jaaskelainen and Palva, 2005; Hynonen and Palva, 2013; Sleytr et al., 2014).

**FIGURE 1:**
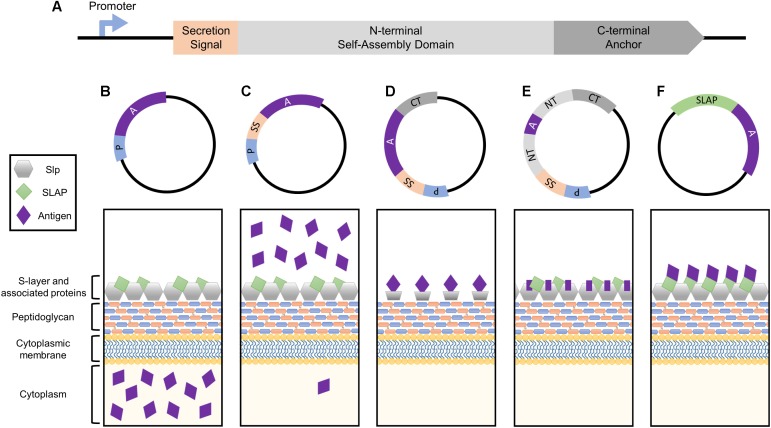
Simplified depiction of the genetic components comprising the Slp protein and how they can be harnessed for heterologous protein expression systems. **(A)** The genetic building blocks of an S-layer gene, including the promoter, secretion signal peptide, N-terminal self-assembly domain, and C-terminal peptidoglycan anchor. Each can be harnessed together or separately for antigen and biotherapeutic delivery applications. **(B)** The Slp promotor induces the production of high levels of target protein which will accumulate within the cytoplasm of the host bacterium. **(C)** The Slp secretion signal yields robust secretion of antigen into the extracellular environment. **(D)** Cell surface display of a foreign peptide can be achieved via fusion to an Slp anchoring domain (N- or C- depending on species). **(E)** The highest levels of antigen expression and cell surface display can be obtained via direct integration into the Slp, but epitope size is extremely limited in order to maintain array formation. **(F)** A novel display strategy utilizing S-layer associated protein (SLAP) fusions which theoretically enable the surface display of much larger proteins.

In the following review, we examine Slp applications in recombinant protein expression and biotherapeutic delivery via their distinct genetic building blocks: the strong native promoter, which can synthesize as many as 500 Slp subunits per second; the signal peptide, that can trigger robust secretion of target molecules; and the structural domains, which can be harnessed for both cell surface display of heterologous proteins or enhancement of host adhesion (**Figure 1A**). Despite the existence of several recombinant protein expression systems based on one or more components of the *Lactobacillus* S-layer, this area of research still remains largely underexploited. Thus, the purpose of this review is to not only shed light on past S-layer studies, but also to advocate for future utilization of Slps in mucosal vaccine and biotherapeutic delivery research.

## Harnessing the Strong Native Promoter

The bacterial S-layer array is composed of an estimated 5 × 10^5^ subunits, representing 10–15% of the total protein content of the bacterium (Sleytr and Messner, 1983; Avall-Jaaskelainen and Palva, 2005). During exponential growth phase, approximately 500 subunits per second must be synthesized, translocated to the cell surface, and incorporated into the existing lattice structure (Sleytr et al., 2014). In order to obtain these high levels of transcription, S-layer genes possess strong, efficient promoters, which can be harnessed for protein production systems (**Figure 1B**). Within the *Lactobacillus* genus, this research has predominately been limited to *slp* promoters of *Lactobacillus acidophilus* and *Lactobacillus brevis*.

Several studies have investigated the versatility of *Lactobacillus* S-layer promoters for driving heterologous protein expression in various LAB hosts. Lactobacilli regularly possess multiple *slp* genes within the same strain that are not all concurrently active (Hynonen and Palva, 2013). Although SlpA is the major constituent of the *L. acidophilus* S-layer, it can be moved to an inactive position triggering expression of the once silent SlpB (Boot et al., 1996a; Konstantinov et al., 2008). When the *L. acidophilus ATCC 4356 slpA* and *slpB* promoters were evaluated in *Lactobacillus casei* ATCC 393, only *slpA* remained active under all tested growth phases (Boot et al., 1996b). However, the same *slpA* promoter, although highly efficient in *L. lactis*, was nearly inactive in isolates of *Lactobacillus reuteri* (Lizier et al., 2010). The *L. acidophilus* NCFM *slpA* promoter was used to drive expression of a green fluorescent protein (GFP) in shuttle vectors based on *oriV1* and *oriV2* replicons (Chen et al., 2014). Similarly, the plasmids exhibited distinct properties based on which strain they were ported into as well as growth-phase-dependent effects. Both plasmids were capable of replicating in strains of *L. casei* and *Lactobacillus delbrueckii*, but only the *oriV1* plasmid, pEL5.6, could replicate in *Lactobacillus paracasei* (Chen et al., 2014). The functionality of the *L. brevis* ATCC 8287 *slpA* promoter was evaluated in three LAB hosts: *L. lactis* MG1614, *Lactobacillus plantarum* NCDO1193 and *Lactobacillus gasseri* NCK334, via the expression of various reporter genes (Kahala and Palva, 1999). The S-layer promoter was recognized in each strain, but was particularly active in *L. lactis* and *L. plantarum.* In fact, aminopeptidase N (PepN) reporter activity within *L. plantarum* was 30-fold higher compared to the *Lactobacillus helveticus* PepN native host and composed a staggering 28% of the total cellular protein during late exponential growth phase. In summary, *Lactobacillus slp* promoters are effective tools for driving recombinant protein expression, but optimization based on host and growth conditions is essential.

The S-layer promoter has also been harnessed for reporter expression *in situ*. Plasmid instability and antibiotic markers can complicate the use of these constructs when moving into human clinical trials or to market, thus chromosomal insertions have gained immense popularity. For *L. acidophilus* NCFM, a pORI-based *upp* counterselective gene replacement system (Goh et al., 2009) has considerably aided this effort (see “Engineering Platforms”). Originally intended for knockout characterizations, it was first employed for a knock-in by cloning a β-glucuronidase (*gusA*) reporter downstream of the *slpA* gene (Douglas and Klaenhammer, 2011). The resulting mutant exhibited a three log increase in GusA activity in comparison to the *gusA*-negative parent, and the study established a framework for the exploitation of highly expressed genomic regions for heterologous protein production (Douglas and Klaenhammer, 2011). Although this particular system was never evaluated within the context of vaccine delivery, the technique was shown effective for expressing antigens using an alternative highly expressed region within the *L. acidophilus* genome (O’Flaherty and Klaenhammer, 2016).

Moving beyond reporter genes and targeting more specific therapeutics, the *L. brevis* JCM 1559 *slpA* promoter was evaluated in an *L. casei* IGM393 host via incorporation into a mouse interleukin 10 (IL-10) secretion system (Kajikawa et al., 2010). Administration of IL-10 was previously shown to be an effective treatment of murine colitis when delivered via recombinant *L. lactis* (Steidler et al., 2000). The authors sought to improve upon this design by substituting in a strain hypothesized to be better adapted to mammalian body temperature. The delivery system was successful in that it yielded high levels of IL-10 secretion when cloned into *L. casei*, but accumulation of the protein varied widely based on pH (Kajikawa et al., 2010). Maximum efficiency occurred at pH 8.0 and dropped drastically as the pH became more acidic. Although the authors attributed low levels of the protein to its physical characteristics (Kajikawa et al., 2010), the selection of a promoter from a free-living species (e.g., *L. brevis*) over a low pH tolerating, vertebrate-adapted organism (e.g., *L. acidophilus*), may have also contributed (Duar et al., 2017). Nonetheless, the S-layer promoter has repeatedly demonstrated its utility for driving protein production systems, and thus merits future research with a focus on therapeutic molecule delivery.

## Exploitation of the Secretion Signal Peptide

All *Lactobacillus* S-layer proteins characterized thus far are preceded by a 25–32 amino acid signal peptide sequence indicative of secretion through the general secretory pathway (Hynonen and Palva, 2013). By cloning this short sequence upstream of an antigen or biotherapeutic molecule, one can obtain robust secretion of a target protein (**Figure 1C**), thus this peptide is frequently harnessed for biotechnological applications.

The *L. brevis* ATCC 8287 *slpA* signal sequence, along with the promoter and transcription terminator, were used to drive expression and secretion of the *Escherichia coli* β-lactamase (*bla*) reporter gene in *L. lactis*, *L. brevis*, *L. plantarum*, *L. gasseri*, and *L. casei*, using a low-copy-number plasmid derived from pGK12 (Savijoki et al., 1997). In all hosts tested, Bla was expressed and released into the culture medium, though highest yields were obtained by recombinant *L. lactis* and the strain of *L. brevis* from which the SlpA components were derived. Production of Bla was mainly restricted to exponential growth phase (Savijoki et al., 1997). Since the system was under control of an *slpA* promoter, it was unsurprising that efficiency was host-specific and growth-phase-dependent (see “Harnessing the strong native promoter”).

The Slp secretion signal has frequently been harnessed for targeted therapeutic applications. In a study seeking to develop a recombinant *L. lactis* mucosal vaccine against porcine post-weaning diarrhea and edema disease, signal peptides from the *L. lactis* major extracellular protein, Usp45, and *L. brevis* SlpA, were used to stimulate the secretion of an *E. coli* F18 fimbrial adhesion protein (FedF) fused to a proteinase PrtP cell wall anchor (Lindholm et al., 2004). Both expression systems induced secretion of all tested FedF-PrtP fusions, however, the quantity of fusion proteins found in the culture medium was four to sixfold higher in those containing the SlpA signal peptide. This was a compelling find considering that the Usp45 signal sequence, previously shown to be one of the most effective *L. lactis* secretion signals (Nouaille et al., 2003), was outperformed by the heterologous SlpA signal sequence. A similar result was obtained with the human interferon alpha 2b (hIFNα-2b) gene (Zhang et al., 2010), used worldwide for the treatment of diseases such as hepatitis B and C (Chelbi-Alix and Wietzerbin, 2007). The addition of the *L. brevis* SlpA signal peptide increased the secretion efficiency threefold in comparison to the lactococcal Usp45 signal sequence (Zhang et al., 2010).

The SlpA signal sequence has also been employed for secretion of chromosomally inserted heterologous proteins. The *Bacillus anthracis* protective antigen (PA), fused to a dendritic cell (DC)-targeting peptide, was previously shown to induce protective immunity when delivered on a plasmid via *L. acidophilus* NCFM (Mohamadzadeh et al., 2009) and *L. gasseri* (Mohamadzadeh et al., 2010). Since a major advantage of using recombinant microbes for vaccine delivery is their ability to express multiple antigens (Wells and Mercenier, 2008), the *B. anthracis* PA was co-expressed with the *Clostridium botulinum* Serotype A neurotoxin heavy-chain antigen (O’Flaherty and Klaenhammer, 2016). The *C. botulinum* vaccine cassette, which utilizes an *L. acidophilus* SlpA secretion signal, was chromosomally inserted downstream of the highly expressed enolase gene. Western blot analysis and RNA sequencing confirmed expression of the two antigens (O’Flaherty and Klaenhammer, 2016). Although this strain was never evaluated *in vivo*, a similarly, constructed strain, also utilizing the SlpA secretion signal, but carrying only the *C. botulinum* antigen (O’Flaherty and Klaenhammer, 2016), was used to vaccinate mice but unable to confer complete protection (Sahay et al., 2018). Rather, vaccine efficacy was enhanced by intradermal injection of the purified immunogenic subunit, which elicited robust memory B cell responses and rendered mice resistant to lethal doses of botulinum neurotoxin serotype A (Sahay et al., 2018).

The Slp signal sequence is capable of stimulating ample secretion of target molecules, prompting its continued use in vaccine research platforms. Although antigen secretion by recombinant LABs has been shown effective for treating disease (Mohamadzadeh et al., 2005, 2009, 2010), exposure to proteolytic enzymes, low pH, and bile salts may encourage protein degradation and therefore decreased functionality (Wells and Mercenier, 2008); consequently cell wall anchoring has become a popular alternative. However, surface display is a balancing act. High exposure of a protein implies optimal host interaction, but also increased susceptibly to degradation. Alternatively, low exposure, via compact protein folding or embedment within the cell wall, confers protection in exchange for diminished efficacy (Michon et al., 2016). Several S-layer-mediated surface display approaches have attempted to resolve this relationship.

## The Self-Assembling and Anchoring Domains of the Slp

Cell wall anchoring via fusion to Slp structural domains (**Figure 1D**) enables direct interaction of target peptides with host mucosal tissues while simultaneously protecting them from degradation (Bermudez-Humaran et al., 2011; Wang et al., 2016). The first S-layer-mediated display of a foreign epitope was generated by fusing the S-layer homology (SLH) domain of *B. anthracis* to the normally secreted levansucrase of *Bacillus subtilis* (Mesnage et al., 1999). The surface-exposed levansucrase retained its enzymatic and antigenic properties, prompting a new area of research which exploits the anchoring abilities of the Slp for cell surface presentation. *Lactobacillus* Slps do not possess SLH domains and are instead composed of two structural domains: a variable terminal for monomer self-assembly (N- or C- depending on the species), and a highly conserved peptidoglycan anchor (Smit et al., 2001; Hynonen and Palva, 2013).

Several studies have used S-layer-mediated anchoring for recombinant protein display on S-layer-deficient bacteria. The cell wall binding domain of the *L. crispatus* F5.7 Slp (LbsA) in conjunction with the Slp promoter and secretion signal, were used for surface display of a GFP on several *Lactobacillus* chicken isolates (Mota et al., 2006). The intention was to generate a vaccine delivery framework for the immunization of broilers against infectious diseases, but never evolved past proof of concept. Vaccination via recombinant bacteria is a particularly attractive option for livestock and poultry operations since the lyophilized microorganisms can be blended into feed; a process that is easily scaled up (Wang et al., 2016). For human applications, the complete SlpA from *L. acidophilus* ATCC 4356 was fused to a GFP reporter for external presentation on a plasmid-cured, lactose-deficient derivative of *L. casei* ATCC 334 (Qin et al., 2014). The authors were able to develop a food-grade cell surface display vector by substituting lactose metabolism genes in place of antibiotic selection markers and verified gastrointestinal stability via *in vitro* modeling (Qin et al., 2014). The *L. crispatus* K2-4-3 SlpB C-terminal domain, LcsB, was employed for the display of not only a GFP reporter (Hu et al., 2011), but also a carcinoembryonic antigen (CEA) (Zhang et al., 2016). Previous studies have shown that CEA is capable of eliciting strong T-cell and humoral immune responses, which can hinder tumor growth (Greiner et al., 2002). Oral administration of recombinant CEA-presenting *L. lactis* to mice yielded significantly higher levels of CEA-specific secretory IgA and a higher spleen index in comparison to CEA antigen alone or the negative control, demonstrating the potential of *L. lactis* CEA as a cancer vaccine (Greiner et al., 2002).

Since the S-layer of *L. crispatus* is capable of binding intestinal extracellular matrices such as collagen and laminin, heterologous expression of Slps has also been used to improve the adhesive capacity of host organisms. For example, the *L. crispatus* JCM5810 collagen-binding S-layer protein, CbsA (Martinez et al., 2000), as well as its individual domains (Antikainen et al., 2002), were expressed on the surface of *L. casei* enabling recombinant organisms to bind various extracellular matrices. Similarly, a surface display cassette consisting of the *L. brevis* ATCC 8287 SlpA receptor-binding domain fused to a PrtP spacer enhanced *L. lactis* adherence to Intestine 407 cells (Avall-Jaaskelainen et al., 2003). Both approaches were able to significantly increase the adhesive capacity of engineered organisms, but yet to be evaluated is the probable synergistic effect of combining improved adhesion with an S-layer anchored antigen in what could potentially be a potent vaccine design platform.

Unlike much of the work presented in this review, a handful of studies achieved Slp-mediated display of foreign proteins without DNA manipulation in the host by exploiting the inherent ability of Slps to anchor and self-assemble. For instance, the *L. acidophilus* C-terminal anchor (SAC) was attached to a GFP reporter, then produced and purified in *E. coli*. The SAC-GFP fusion protein was capable of binding lithium chloride-pretreated surfaces of wild-type *L. acidophilus*, *L. helveticus*, and *L. crispatus* (Smit et al., 2001). Similarly, *L. crispatus* K2-4-3 LcsB-GFP fusions were able to associate with SDS-pretreated surfaces of various S-layer-forming LABs including *L. brevis*, *L. helveticus*, *L. crispatus*, and *Lactobacillus salivarius* as well as several non-S-layer-formers, including *L. lactis, L. delbrueckii*, *Lactobacillus johnsonii*, and *Streptococcus thermophilus* (Hu et al., 2011). Interestingly, neither the SAC-GFP fusion nor LcsB-GFP were able to bind the surface of *L. casei* (Smit et al., 2001; Hu et al., 2011). This approach is unique in that it offers an alternative way to deliver foreign proteins while also circumventing the GMO (genetically modified organism) label, but is limited by its inability to generate additional heterologous protein *in vivo* and susceptibility to replacement by wild type Slps. However, in general, studies using heterologously expressed Slps and Slp anchors are hindered by inadequate secretion across the cell wall (Hu et al., 2011) or inability to form an array due to irregular folding and/or lack of cell surface exposure (Martinez et al., 2000). Consequently, rather than tease apart the efficient Slp secretion and display system, there is now interest in harnessing it as a whole through the direct insertion of foreign peptides within the context of the protein.

## Delivery Via Direct Integration Within the Slp

The extraordinarily high, stable abundance of the Slp, makes it an enticing target for antigen display and delivery via direct integration into its genome sequence (**Figure 1E**). The presentation of an exogenous protein within the context of the Slp was first achieved in *Caulobacter crescentus* through the random introduction of a pilin peptide from *Pseudomonas aeruginosa* strain K. Eleven potential sites of successful insertion were identified, demonstrating for the first time the capacity of the Slp to act as a carrier for foreign epitopes (Bingle et al., 1997).

Many subsequent studies have focused on mapping the S-layer to gain insight into ideal positioning for novel insertions, including in *Lactobacillus*. In *L. acidophilus* ATCC 4356, peptides ranging from 7 to 13 amino acids were randomly introduced into the Slp (Smit et al., 2002). Within the variable N-terminal (SAN), five of these positions maintained paracrystalline structure formation *in vitro*, while four others resulted in the complete abolishment of any array-forming capacity. Unsurprisingly, an insertion into the cell wall-binding domain had no effect on assembly (Smit et al., 2002). Similarly, the *L. brevis* SlpA was mapped via cysteine-scanning mutagenesis combined with sulfhydryl modification to identify locations of high surface accessibility and verify that the mutations did not alter self-assembly properties (Vilen et al., 2009). Combined, these works established several stable, surface-accessible insertion sites within the *Lactobacillus* Slp, yet few researchers have capitalized on this knowledge.

Currently, only two studies have successfully integrated antigens within the context of the *Lactobacillus* Slp. Through an inducible expression system, the poliovirus VP1 epitope was evaluated in four potential *L. brevis* ATCC 8287 *slpA* insertion sites (Avall-Jaaskelainen et al., 2002). The location that demonstrated the best surface expression was then targeted for chromosomal insertion of the c-Myc epitope via direct double-crossover integration (Avall-Jaaskelainen et al., 2002). A uniformly chimeric S-layer was obtained without any effect on array formation. More recently, the membrane proximal external region (MPER) epitope from human immunodeficiency virus type 1 (HIV-1) was inserted into *L. acidophilus* NCFM SlpA (Kajikawa et al., 2015). *L. acidophilus* NCFM is regularly employed for mucosal vaccine delivery due in part to its direct interactions with the dendritic cell-specific antigen DC-SIGN (Konstantinov et al., 2008) and adaptation to the harsh conditions associated with gastric transit (Sanders and Klaenhammer, 2001). Vaccination via the recombinant organism, in conjunction with an IL-1β adjuvant, successfully stimulated MPER-specific antibody production in both the serum and mucosal secretions of mice (Kajikawa et al., 2015). This study marks the first and only instance of an Slp-integrated antigen being evaluated *in vivo*.

The establishment of a uniformly chimeric S-layer translates to approximately 10^5^ instances of epitope display on the surface of a single bacterium (Sleytr and Messner, 1983). Despite these considerable numbers, insert size is exceptionally limited in order to preserve S-layer array formation (Smit et al., 2002). Currently, peptides longer than 19 amino acids are unable to be inserted into SlpA without disrupting the lattice structure (Kajikawa et al., 2015). Therefore, alternative methods exploiting auxiliary proteins associated with the S-layer are now being investigated for the display and delivery of larger antigens, as seen in Slp-mediated anchoring studies (see “The self-assembling and anchoring domains of the Slp”), but at frequencies more akin to direct integration.

## S-Layer Associated Proteins

The *Lactobacillus* S-layer, once thought to be solely composed of repeating monomeric Slp subunits, is actually far more complex (Johnson et al., 2013, 2015). It is now widely accepted that S-layers can act as scaffolds for the external display of numerous auxiliary proteins, termed S-layer associated proteins (SLAPs), which can confer additional physiological functionalities (Johnson et al., 2013, 2015, 2017; Hymes et al., 2016; Johnson and Klaenhammer, 2016; Celebioglu and Svensson, 2017). Recently, the SLAP profile of *L. acidophilus* NCFM was quantified via multiplexing mass spectrometry (Klotz et al., 2017). Although results revealed significant growth stage-dependent alterations, they also highlighted several proteins with consistent high expression in both logarithmic and stationary growth phases (Klotz et al., 2017). The surface location and abundance of these proteins make them excellent targets for biotherapeutic delivery. Unlike Slp integrants, SLAP fusions are theoretically less limited in epitope size as they are not prone to S-layer array disruption (**Figure 1F**). Both the native SLAP promoter and secretion signal can be harnessed for this process with the intent to maintain high expression, secretion, and surface localization coupled with the display and delivery of a significantly larger and therefore more potent epitope.

## Slp Engineering Platforms

Presently, the two most popular techniques for engineering LABs are the NICE (NIsin Controlled gene Expression) system in *L. lactis* and the pORI-based *upp* counterselective gene replacement system in *L. acidophilus.* There is also growing interest surrounding the use of CRISPR-Cas technology, though its application in bacterial genome editing remains relatively underrepresented (Selle and Barrangou, 2015; Hidalgo-Cantabrana et al., 2017).

### The NICE System

The NICE system uses nisin to drive heterologous protein expression in *L. lactis* (Kuipers et al., 1995). Through the insertion of signal transduction genes from a nisin gene cluster into a nisin-negative *L. lactis* strain, NZ9000 was created (Kuipers et al., 1998). Subsequently, when a gene of interest is inserted downstream of the inducible *nisA* promoter, expression of that gene can be obtained by the addition of nisin to the culture medium (Mierau and Kleerebezem, 2005). Since its conception, NICE has become one of the most successful and widely used expression systems in Gram-positive bacteria (Mierau and Kleerebezem, 2005). Indeed, the NICE system was even employed for the production a number of the S-layer fusion proteins mentioned above (Hu et al., 2011; Zhang et al., 2016) and to render the non-adhesive *L. lactis* NZ9000 adhesive via the addition of an *L. brevis* SlpA receptor-binding domain (Avall-Jaaskelainen et al., 2003). The availability of an easily engineered, non-S-layer-forming organism, has greatly accelerated not only our understanding of the biological role of an S-layer but also how we can exploit it.

### The pORI-Based *upp* System

The establishment of the pORI-based *upp* counterselective gene replacement system in S-layer-former *L. acidophilus* NCFM, first employed for the functional characterization of SlpX (Goh et al., 2009), has since become an invaluable tool for S-layer component engineering. The system uses a *upp*-encoded uracil phosphoribosyltransferase (UPRTase) as a counterselection marker to positively select for double crossover homologous recombination events. The method has been adapted for numerous Slp studies ranging from reporter integration (Douglas and Klaenhammer, 2011), anchoring/adjuvant assessment (Kajikawa et al., 2011), and targeted antigen delivery systems for disease protection (Kajikawa et al., 2015; O’Flaherty and Klaenhammer, 2016). Similar counterselective systems have also been developed in non-S-layer-formers including *L. gasseri* ATCC 33323 (Selle et al., 2014) and *L. casei* ATCC 393 (Song et al., 2014), but have yet to be harnessed for Slp analyses. In general, the technique remains a superior approach for characterizing the functional genetics of lactobacilli without the additional pressures required for plasmid maintenance.

### CRISPR

**FIGURE 2:**
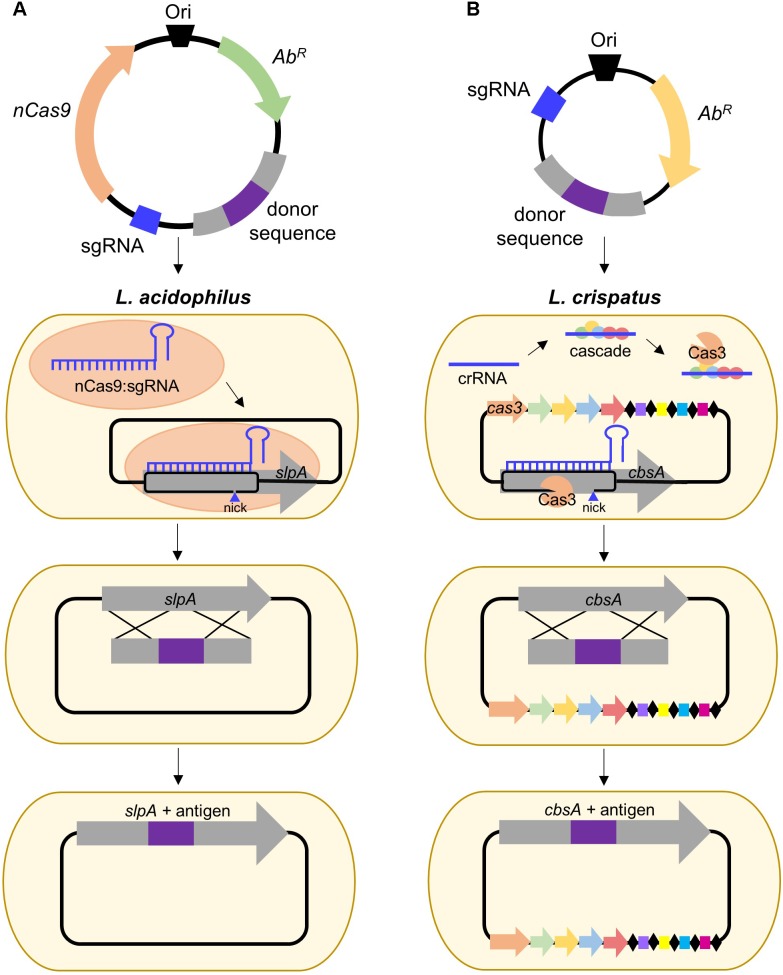
Proposed strategies for CRISPR-based genome editing in relevant S-layer-forming lactobacilli. **(A)** Antigen integration into *L. acidophilus* SlpA via an exogenous Type II system employing a Cas9 nickase variant which introduces a guide RNA-targeted single-stranded break. Unlike wild-type Cas9 which generates blunt double-stranded breaks (DSB), nickases cut only one strand of the DNA, permitting genome editing in bacteria deficient in DSB repair (Chiang et al., 2016; Song et al., 2017). **(B)** Antigen integration into *L. crispatus* CbsA using the endogenous Type I system which consists of the CRISPR-associated complex for antiviral defense (Cascade) and the signature Cas3 nuclease (Barrangou, 2015).

Clustered Regularly Interspaced Short Palindromic Repeats (CRISPR) together with CRISPR-associated (Cas) proteins, form the prokaryotic adaptive immune system which provides DNA-encoded, RNA-guided, sequence-specific protection against viral invaders (Barrangou and Doudna, 2016). The current classification system uses two broad classes consisting of six major types (I–VI) which can be allocated into approximately 30 subtypes (Klompe and Sternberg, 2018). The simplest of these is the Type II system which relies on the activity of a singular Cas9 endonuclease and has gained immense popularity for its ability to be repurposed for genome editing (Chylinski et al., 2014; Barrangou and Doudna, 2016; Klompe and Sternberg, 2018). Depending on the organism, this can be done via harnessing of the endogenous system or supplying an exogenous Cas9:single guide RNA (sgRNA) complex. To date, no CRISPR-based genome editing has been performed in S-layer-forming lactobacilli, but it has been conducted in *L. casei* (Song et al., 2017) and *L. lactis* (Berlec et al., 2018), frequent hosts for recombinant Slp investigative research. Nevertheless, the development and delivery of a functional CRISPR-Cas9 plasmid remains a critical need, particularly in organisms such as *L. acidophilus* NCFM (depicted in **Figure 2A**), which does not possess an active CRISPR system (Crawley et al., 2018), but has proven to be hugely impactful in both probiotic and Slp research.

Despite the popularity of Type II engineering, Type I systems have been harnessed for genome editing in *Clostridium* and archaea (Pyne et al., 2016; Cheng et al., 2017) and transcriptional regulation in *E. coli* (Luo et al., 2015). S-layer formers, *L. crispatus*, and *L. helveticus*, frequently possess Type I systems (Crawley et al., 2018) and are also of interest for vaccine delivery. *L. crispatus* is a natural inhabitant of the human vaginal tract (Lepargneur, 2016), rendering it suitable for delivery of antigens targeting sexually transmitted diseases, such as HIV, while *L. helveticus* is predominately associated with dairy (Taverniti and Guglielmetti, 2012), which could be advantageous when considering modes of delivery and stability of the vaccine. A proposed strategy for Type I genome editing in *L. crispatus* is illustrated in **Figure 2B**. Harnessing of the endogenous system or delivery of a functional exogenous system both possess the potential to be powerful tools for advancing Slp-mediated biotherapeutic research.

The NICE and pORI-based *upp* systems have both proven effective for S-layer-mediated biotherapeutic delivery; however, next-generation genome editing tools, such as CRISPR-Cas, hold tremendous potential for bacterial engineering overall (Selle and Barrangou, 2015; Hidalgo-Cantabrana et al., 2017). Many S-layer-forming lactobacilli possess endogenous CRISPR systems, making them promising candidates for future S-layer engineering studies. Alternatively, delivery of a functional CRISPR to strains devoid of a system, will also greatly accelerate the pace at which recombinant organisms can be generated. Ironically, despite CRISPR originating as the bacterial adaptive immune system, CRISPR-based bacterial genome editing still remains relatively underexploited, though recent studies have provided valuable insights for its widespread future implementation (Selle and Barrangou, 2015).

## Future Directions

The Slp is a multi-faceted engineering target with both biotherapeutic and biotechnological applications (Sleytr et al., 2014). However, within the *Lactobacillus* genus, harnessing of this protein remains early in its development (Avall-Jaaskelainen and Palva, 2005; Hynonen and Palva, 2013); nonetheless, the research highlighted above (summarized in **Table 1**) advocates for its continued pursuit. The *slp* promoter and signal peptide are undoubtedly adept at driving robust expression and secretion of target proteins, while the structural domains have successfully displayed foreign epitopes and improved the adhesive capacity of host cells. More complex and novel display strategies, such as direct integration into the Slp or SLAP fusions, are innovative approaches for cell surface presentation that also exploit the inherent properties of S-layer-forming lactobacilli. In general, eliciting consistent immune responses via the mucosal route of administration is hindered by rapid elimination or inability to make contact with M cells and other mucosal tissues involved in antigen uptake and processing (Ogra et al., 2001). Thus the ability of recombinant LABs, and the S-layer in particular, to promote antigen uptake and stimulate the adaptive immune response is highly desirable (Konstantinov et al., 2008).

**Table 1:** S-layer protein applications in recombinant protein expression systems and biotherapeutic delivery platforms.

S-layer-forming lactobacilli	Slp	Slp Component	Host	Antigen/Reporter	Results	Reference
*L. acidophilus*	SlpA; SlpB	Promoter	*L. casei*	CAT	Evaluated *slpA* and *slpB* promoters; only *slpA* remained active under all tested growth conditions	Boot et al., 1996b
	SlpA	Promoter	*L. lactis; L. reuteri* chicken crop isolates	eGFP	The *slpA* promoter was highly efficient in *L. lactis* but nearly inactive in *L. reuteri* isolates	Lizier et al., 2010
	SlpA	Promoter	*L. casei; L. paracasei; L. plantarum; L. lactic; L. helveticus; L. acidophilus; L. lactis; E. coli*	GFP	Plasmids encoding *slpA* promoter exhibited distinct properties based on host and growth phase	Chen et al., 2014
	SlpA	Promoter	*L. acidophilus*	GusA	The *slpA*-driven GusA activity increased three logs in comparison to the *gusA*-negative parent	Douglas and Klaenhammer, 2011
	SlpA	Secretion	*L. acidophilus*	*B. anthracis* protective antigen (PA); *C. botulinum* Serotype A neurotoxin heavy-chain antigen	The SlpA signal sequence generated stable and robust secretion of the *C. botulinum* antigen	O’Flaherty and Klaenhammer, 2016
	SlpA	Secretion	*L. acidophilus*	*C. botulinum* Serotype A neurotoxin heavy-chain antigen	Recombinant organism was unable to confer complete protection against an experimental botulism challenge	Sahay et al., 2018
	SlpA	Integration	*L. casei*	GFP	Generated a food-grade SlpA-based cell surface display vector and verified gastrointestinal stability *in vitro*	Qin et al., 2014
	SlpA	Integration	*L. acidophilus*	HIV-1 membrane proximal external region (MPER)	Delivery of MPER peptide via direct integration into SlpA stimulated antigen-specific antibody production in both serum and mucosal secretions of vaccinated mice	Kajikawa et al., 2015
*L. brevis*	SlpA	Promoter	*L. lactis; L. plantarum; L. gasseri*	GusA; Luc; PepN	The *slpA* promoter was recognized in all strains but especially *L. lactis* and *L. plantarum*	Kahala and Palva, 1999
	SlpA	Promoter	*L. casei*	Mouse IL-10	The *slpA* promoter yielded high levels of IL-10 but was sensitive to low pH	Kajikawa et al., 2010
	SlpA	Promoter; secretion	*L. lactis; L. brevis; L. plantarum; L. gasseri; L. casei*	*E. coli* β-lactamase (Bla)	Bla was expressed in all hosts, but most efficiently in *L. lactis* and *L. brevis*; production was restricted to exponential growth phase	Savijoki et al., 1997
	SlpA	Secretion	*L. lactis*	*E. coli* F18 fimbrial adhesion protein (FedF)	The SlpA signal sequence increased FedF secretion efficiency four to sixfold in comparison to the lactococcal Usp45 signal sequence	Lindholm et al., 2004
	SlpA	Secretion	*L. lactis*	Human interferon alpha 2b (hIFNα-2b)	SlpA signal sequence increased hIFNα-2b secretion efficiency threefold in comparison to the lactococcal Usp45 signal sequence	Zhang et al., 2010
	SlpA	Secretion; structural domain	*L. lactis*	None	Surface expression of SlpA receptor-binding domain increased adherence to Intestine 407 cells	Avall-Jaaskelainen et al., 2003
	SlpA	Integration	*L. brevis*	Poliovirus VP1 epitope; c-Myc epitope	Directly inserted epitopes into SlpA without disrupting array formation	Avall-Jaaskelainen et al., 2002
*L. crispatus*	LbsA	Promoter; secretion, structural domain	*Lactobacillus* chicken isolates	GFP	Achieved expression, secretion and surface presentation of GFP	Mota et al., 2006
	LcsB	Structural domain	*L. lactis*	GFP	Achieved surface presentation of GFP	Hu et al., 2011
	LcsB	Structural domain	*L. lactis*	Carcinoembryonic antigen (CEA)	LcsB-mediated display of CEA stimulated higher levels of antigen-specific secretory IgA and a higher spleen index when fed to mice	Zhang et al., 2016
	CbsA	Structural domain	*L. casei*	None	Recombinant *L. casei* expressing CbsA was able to bind immobilized collagens	Martinez et al., 2000
	CbsA	Structural domain	*L. casei*	None	Recombinant expression of CbsA domains enabled adhesion to laminin and collagen	Antikainen et al., 2002

Numerous studies have established vaccine platforms based on one or more components of the *Lactobacillus* Slp, however, there remains a disconnect between delivery and efficacy. Despite extensive reviews touting the effectiveness of the LAB-based vaccines *in vivo* (Wells and Mercenier, 2008; LeCureux and Dean, 2018), few S-layer-based delivery frameworks have moved into animal models. Although successful secretion and/or surface display of the reporter/antigen is regularly achieved, only three of the recombinant organisms presented above were tested in mice (Kajikawa et al., 2015; Zhang et al., 2016; Sahay et al., 2018), whereas the remainder were more focused on establishing that antigen production/display was even possible. Thus, an important step moving forward will be connecting the delivery of these antigens with an actual vaccination event, therefore surpassing proof of concept studies and ultimately demonstrating disease protection.

## Author Contributions

CK and RB wrote and edited the manuscript.

## Conflict of Interest Statement

The authors declare that the research was conducted in the absence of any commercial or financial relationships that could be construed as a potential conflict of interest.
